# Global Myocardial Wall Thickness in Transfusion-Dependent Thalassemia: A Cross-Sectional MRI Analysis

**DOI:** 10.3390/diagnostics15212805

**Published:** 2025-11-05

**Authors:** Antonella Meloni, Laura Pistoia, Giuseppe Peritore, Michela Zerbini, Stefania Renne, Priscilla Fina, Antonino Vallone, Filomena Longo, Anna Spasiano, Zelia Borsellino, Valerio Cecinati, Giuseppe Messina, Elisabetta Corigliano, Vincenzo Positano, Andrea Barison, Alberto Clemente

**Affiliations:** 1 Bioengineering Unit, Fondazione G. Monasterio CNR-Regione Toscana, 56124 Pisa, Italy; positano@ftgm.it; 2 Unità Operativa Complessa Ricerca Clinica, Fondazione G. Monasterio CNR-Regione Toscana, 56124 Pisa, Italy; 3 Unità Operativa Complessa di Radiologia, Azienda di Rilievo Nazionale ad Alta Specializzazione Civico “Benfratelli-Di Cristina”, 90127 Palermo, Italy; giuseppe.peritore@hotmail.it; 4 Diagnostica per Immagini e Radiologia Interventistica, Ospedale del Delta, 44023 Lagosanto (FE), Italy; m.zerbini@ausl.fe.it; 5 Struttura Complessa di Cardioradiologia-UTIC, Presidio Ospedaliero “Giovanni Paolo II”, 88046 Lamezia Terme (CZ), Italy; stefania.renne@virgilio.it; 6 Unità Operativa Complessa Diagnostica per Immagini, Ospedale “Sandro Pertini”, 00157 Roma, Italy; priscilla.fina@gmail.com; 7 Reparto di Radiologia, Azienda Ospedaliera “Garibaldi” Presidio Ospedaliero Nesima, 95126 Catania, Italy; ninovallone@hotmail.com; 8 Dipartimento di Medicina Specialistica, Day Hospital Della Talassemia e Delle Emoglobinopatie, Azienda Ospedaliero-Universitaria Arcispedale “S. Anna”, 44124 Cona (FE), Italy; filomena.longo@ospfe.it; 9 Unità Operativa Semplice Dipartimentale Malattie Rare del Globulo Rosso, Azienda Ospedaliera di Rilievo Nazionale “A. Cardarelli”, 80131 Napoli, Italy; spasiano.anna@tiscali.it; 10 Unità Operativa Complessa Ematologia con Talassemia, Azienda di Rilievo Nazionale ad Alta Specializzazione Civico “Benfratelli-Di Cristina”, 90127 Palermo, Italy; zelia.borsellino@arnascivico.it; 11 Struttura Semplice di Microcitemia, Ospedale “SS. Annunziata”, 74123 Taranto, Italy; valerio.cecinati@asl.taranto.it; 12 Centro Microcitemie, Grande Ospedale Metropolitano “Bianchi-Melacrino-Morelli”, 89100 Reggio Calabria, Italy; gspmessina@virgilio.it; 13 Ematologia Microcitemia, Ospedale San Giovanni di Dio, ASP Crotone, 88900 Crotone, Italy; elisabetta.corigliano@asp.crotone.it; 14 Cardiology and Cardiovascular Medicine, Fondazione G. Monasterio CNR-Regione Toscana, 56124 Pisa, Italy; abarison@ftgm.it; 15 Department of Radiology, Fondazione G. Monasterio CNR-Regione Toscana, 56124 Pisa, Italy; clemente@ftgm.it

**Keywords:** transfusion-dependent thalassemia, global wall thickness index, cardiovascular magnetic resonance, heart failure

## Abstract

**Background:** This retrospective cross-sectional study evaluated the association of the global wall thickness index (GTI), derived from cardiovascular magnetic resonance (CMR), with demographic, clinical, and imaging findings, as well as heart failure history in transfusion-dependent thalassemia (TDT) patients. **Methods:** We analyzed 1154 TDT patients (52.9% female, 37.46 ± 10.67 years) from the Extension-Myocardial Iron Overload in Thalassemia project and 167 healthy controls (54.5% female, 36.33 ± 15.78 years). The CMR protocol included the T2* technique for the assessment of iron overload, cine imaging for the assessment of left ventricular (LV) function and size, and late gadolinium enhancement (LGE) imaging for the detection of replacement myocardial fibrosis (in the subset of 366 patients who underwent contrast administration). GTI (in mm/m^2^) was calculated from LV mass and end-diastolic volume. **Results:** GTI discriminated TDT patients from controls better than the LV end-diastolic volume index. Among TDT patients, GTI was higher in males, in those with diabetes, and in those with severe myocardial iron overload (cardiac T2* < 10 ms), but was unrelated to age, hemoglobin and ferritin levels, splenectomy, hepatic and pancreatic T2* values, LV ejection fraction, and fibrosis. GTI showed a diagnostic performance comparable to global heart T2* and superior to LV ejection fraction in identifying patients with prior heart failure. **Conclusions:** GTI is elevated in TDT patients compared with healthy controls. Male sex and severe myocardial iron overload are key determinants of GTI in TDT. Increased GTI is linked to a history of heart failure, supporting its role as a complementary tool to conventional CMR indices.

## 1. Introduction

Transfusion-dependent thalassemia (TDT) represents the most severe clinical phenotype of thalassemia syndromes, caused by genetic defects in hemoglobin synthesis, leading to ineffective erythropoiesis and severe anemia [1,2,3]. Lifelong, regular red blood cell transfusions remain the cornerstone of management, ensuring adequate oxygen delivery and promoting normal growth and development [4,5,6]. These advances, combined with iron chelation therapy, have transformed TDT from a fatal childhood disease into a chronic condition compatible with adulthood [7,8]. Despite these improvements, transfusion therapy inevitably leads to progressive iron accumulation, particularly in the liver, endocrine glands, and heart [9,10,11]. Myocardial iron overload (MIO) is the main determinant of morbidity and mortality in TDT [12,13,14], leading to oxidative stress, cellular damage, and the activation of inflammatory processes [15,16,17]. These processes contribute to the development of a cardiomyopathy, which typically manifests in two principal phenotypes [17,18,19,20]. The more common dilated form is characterized by progressive left ventricular (LV) dilation and systolic dysfunction, whereas the restrictive form is associated with impaired compliance and diastolic dysfunction, often leading to elevated filling pressures. Both pathways may progress to overt heart failure [18,20]. In addition to iron toxicity, chronic anemia contributes to cardiac remodeling [19]. Reduced oxygen-carrying capacity triggers compensatory mechanisms, including increased cardiac output, LV dilation, and eccentric hypertrophy [21,22,23], that can culminate in high-output heart failure [19]. Furthermore, as survival improves, comorbidities such as endocrine dysfunction (e.g., diabetes, hypogonadism, and hypothyroidism) and aging-related changes increasingly contribute to cardiac risk in this population [24,25,26]. The convergence of these stressors produces a heterogeneous spectrum of myocardial remodeling, underscoring the importance of a comprehensive cardiac assessment.

Cardiac magnetic resonance (CMR) imaging is the reference standard for noninvasive assessment of myocardial iron levels [27,28] and of ventricular morphology and function [29,30]. Conventional indices such as LV ejection fraction (EF), mass, and volume are crucial for risk stratification in a wide range of diseases [31,32,33,34,35], including TDT [36], but they often become abnormal only at advanced stages, leaving earlier structural changes in wall thickness, geometry, or myocardial mechanics undetected [37,38]. Novel CMR-derived markers, including strain and myocardial contraction fraction (MCF), have improved early detection of myocardial involvement and prognostic stratification in TDT [39,40] and across diverse disorders [41,42,43,44,45].

Global wall thickness (GT) and its body surface area-indexed counterpart, the global wall thickness index (GTI), have recently emerged as simple, reproducible parameters that integrate LV mass and end-diastolic volume into a single metric, offering a robust global estimate of average myocardial wall thickness [46]. A large CMR study demonstrated that GT independently predicts adverse outcomes, including death and heart failure hospitalization, while GTI can identify at-risk individuals even with preserved LV mass and EF [46]. Their high sensitivity to subtle wall thickness changes, combined with reliance on standard CMR metrics, makes GT and GTI valuable tools for early detection and routine monitoring of myocardial remodeling.

Given the complex and multifactorial nature of cardiac remodeling in TDT, GTI may represent a particularly valuable tool. However, its clinical role in this population remains unexplored.

Against this background, the present multicenter study sought to (1) characterize the distribution of GTI in TDT patients compared with healthy controls; (2) examine cross-sectional associations between GTI and demographic, clinical, and laboratory characteristics in TDT; (3) assess the relationship of GTI with myocardial iron overload, ventricular function, and fibrosis; (4) evaluate the potential clinical utility of GTI as a risk marker for heart failure in TDT.

## 2. Materials and Methods

### 2.1. Study Population

We retrospectively selected all 1154 patients with TDT (52.9% females, mean age 37.46 ± 10.67 years) who had been consecutively enrolled in the Extension–Myocardial Iron Overload in Thalassemia (E-MIOT) project in the nine-year interval between 2015 and 2024. The E-MIOT network is a nationwide Italian consortium comprising 66 thalassemia care centers and 15 magnetic resonance (MRI) units adhering to standardized and validated MRI protocols [47]. All centers are linked via a web-based database that records demographic, clinical, and imaging data for all patients.

All TDT patients had been receiving regular red blood cell transfusions since early childhood to maintain pre-transfusion hemoglobin levels above 9–10 g/dL. MRI assessments were scheduled within one week before a routine transfusion. Chelation therapy was introduced in the mid-to-late 1970s for older patients, while individuals born after this period generally began chelation during early childhood.

The control group consisted of 167 healthy individuals (54.5% females, mean age 36.32 ± 15.78 years) recruited from a multicenter cohort established to determine reference values for cardiac functional parameters and myocardial T1 values [48]. Control subjects were eligible if they had a normal resting electrocardiogram, no history or clinical evidence of cardiovascular disease, no conventional cardiovascular risk factors or systemic disorders, and no absolute contraindications to MRI.

The study adhered to the ethical principles outlined in the Declaration of Helsinki and received approval from the appropriate institutional ethics committee. All participants provided written informed consent.

### 2.2. MRI

MRI examinations were conducted on standard 1.5T clinical scanners from three vendors (Signa Excite HD or Artist, GE Healthcare, Milwaukee, WI, USA; Ingenia or Achieva, Philips, Best, The Netherlands; MAGNETOM Sola or Aera, Siemens, Erlangen, Germany). All acquisitions were performed during end-expiratory breath-holding with electrocardiographic gating.

T2* gradient-echo multiecho sequences [10 echo times (TEs), echo spacing 2.26 ms] were acquired to assess tissue iron overload. A mid-transverse hepatic slice [49], at least five axial slices covering the entire pancreas [50], and three short-axis LV views (basal, mid, and apical) [51] were obtained. T2* image analysis was performed by experienced (>15 years) operators using HIPPOMIOT^®^ (Version 2.0, Consiglio Nazionale delle Ricerche and Fondazione Toscana Gabriele Monasterio, Pisa, Italy), a custom-written and previously validated software. Hepatic T2* values were measured within a circular region of interest (ROI) [49] and subsequently converted into liver iron concentration (LIC) values [52]. For the pancreas, three small ROIs were manually placed over the head, body, and tail, carefully restricted to parenchymal tissue while avoiding major vessels, ducts, and regions prone to susceptibility artifacts from adjacent structures such as the stomach or colonic lumen [50]. A global pancreatic T2* value was derived as the mean of these three measurements. Myocardial T2* values were analyzed using a 16-segment LV model, following the American Heart Association/American College of Cardiology standardized segmentation guidelines [53]. To account for cardiac and visceral geometric distortions as well as susceptibility artifacts, an internal correction map within the software was applied. The global heart T2* value was then calculated as the average of all segmental values. The reproducibility of T2* measurements across sites, studies, and observers had been previously assessed [47].

To quantify LV function parameters, steady-state free precession (SSFP) cine images were acquired during 8 s breath-holds in the vertical and horizontal long-axis planes, as well as in sequential contiguous 8 mm short-axis slices covering the ventricles from the atrioventricular ring to the apex [54]. Thirty cardiac phases were acquired per heartbeat. Imaging parameters included: flip angle 45°, TE 1.6 ms, repetition time 3.7 ms, and matrix size 192 × 192 pixels. Image analysis was performed by experienced operators, blinded to clinical data, using a commercial clinical workstation (MASS software, Medis, Leiden, The Netherlands, or cmr42, Circle Cardiovascular Imaging Inc., Calgary, AB, Canada). Endocardial and epicardial borders were manually traced in the short-axis stack at end-diastole and end-systole and end-diastolic and end-systolic volumes (EDV and ESV) were calculated without relying on a specific ventricular geometry. LV mass (LVM) was determined by multiplying myocardial volume by the specific myocardial density, assumed to be 1.05 g/cm^3^. LV volumes and mass were indexed to body surface area (BSA), calculated using a modified Dubois and Dubois formula [55]. Inter-center variability for quantification of LV functional parameters within the E-MIOT network has been previously determined [56]. The mean LV end-diastolic global wall thickness was quantified using the optimized equation [46]:

GT = 0.05 + 1.60 * LVM^0.84^ * LVEDV^−0.49^

GTI, expressed in mm/m^2^, was calculated as the ratio between GT and BSA.

Replacement/focal myocardial fibrosis was assessed by late gadolinium enhancement (LGE) imaging using a T1-weighted gradient-echo inversion-recovery sequence [flip angle 20°, minimum TE, inversion times 250–300 ms, matrix 224 × 192 pixels, slice thickness 8 mm]. Short-axis and long-axis views were acquired 8–18 min after intravenous administration of Gadobutrol (Gadovist^®^, Bayer Schering Pharma, Berlin, Germany) at a standard dose of 0.2 mmol/kg body weight. LGE imaging was not performed in patients with a glomerular filtration rate (GFR) < 30 mL/min/1.73 m^2^ or in those who refused the injection of contrast medium. Images were qualitatively assessed for the presence, pattern, and regional distribution of LGE [36]. LGE was confirmed only when observed in at least two imaging planes. Patterns were classified as ischemic when enhancement was subendocardial or transmural within a coronary artery territory; all other patterns were categorized as non-ischemic. The extent of LGE was assessed semi-quantitatively by estimating the number of affected myocardial segments.

### 2.3. Biochemical Assays

Biochemical tests were performed at the local laboratories of each participating thalassemia center using commercially available, standardized assay kits. For analysis, the average hemoglobin and serum ferritin values over the 12 months prior to the MRI examination were calculated for each patient.

### 2.4. Diagnostic Criteria

A threshold of 20 ms was adopted as a conservative cut-off for both segmental and global myocardial T2* values [57]. Patients with MIO were further categorized as having severe MIO (T2* < 10 ms) or moderate-to-mild MIO (T2* between 10 and 20 ms).

Diabetes mellitus was diagnosed in accordance with standard criteria: fasting plasma glucose ≥ 126 mg/dL, 2 h plasma glucose ≥ 200 mg/dL during an oral glucose tolerance test (OGTT), or random plasma glucose ≥ 200 mg/dL in the presence of classic hyperglycemia symptoms or a hyperglycemic crisis [58].

Heart failure was diagnosed based on a combination of clinical symptoms (e.g., fatigue, shortness of breath, ankle swelling), physical signs, biomarker levels, and imaging or other instrumental findings, following current guideline recommendations [59].

### 2.5. Statistical Analysis

Statistical analysis was conducted using SPSS version 27.0 (IBM Corp, Armonk, NY, USA) and MedCalc version 19.8 (MedCalc Software Ltd., Ostend, Belgium) statistical packages.

Continuous variables are expressed as mean ± standard deviation (SD) and categorical variables as frequencies and percentages.

The Kolmogorov–Smirnov test was used to assess the normality of continuous variables.

For continuous variables with a normal distribution, comparisons between groups were performed using the independent-samples *t*-test (for two groups) or one-way ANOVA (for more than two groups). For continuous variables with a non-normal distribution, the Mann–Whitney *U* test or Kruskal–Wallis test was applied. Categorical variables were compared using the χ^2^ test. The Bonferroni post hoc test was used for multiple comparisons between pairs of groups.

Correlations were assessed using Pearson’s or Spearman’s tests.

Univariable regression analyses were first performed to explore potential determinants of GTI. Variables showing statistical significance (*p* < 0.05) were subsequently included in a stepwise multivariable regression model to identify independent predictors. Multicollinearity of variables tested in the multivariate model was assessed using the variance inflation factor (inflated if > 5) and its tolerance statistic (inflated if < 0.20).

Receiver operating characteristic (ROC) curve analysis was used to evaluate diagnostic performance and determine optimal cut-off values for predicting a specific condition. The area under the curve (AUC) with 95% confidence intervals (CIs), sensitivity, and specificity were calculated. The DeLong’s test was applied to assess whether differences in AUCs between parameters were statistically significant.

All tests were two-sided, and *p* < 0.05 was considered statistically significant.

## 3. Results

### 3.1. Comparison Between TDT Patients and Healthy Subjects

**Figure 1:**
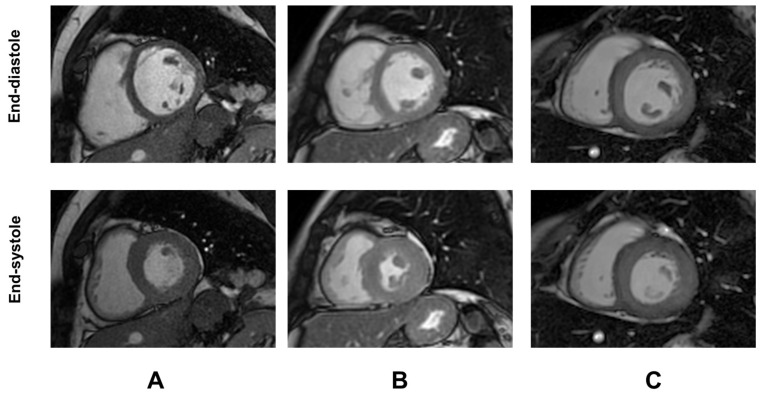
Representative examples of global wall thickness index (GTI) in three female patients with TDT. (**A**) Patient with normal GTI. (**B**) Patient with increased GTI but normal LV mass index [concentric remodeling]. (**C**) Patient with both increased GTI and LV mass index [concentric hypertrophy].

Demographic and clinical characteristics and MRI findings of TDT patients are summarized in Table 1. Representative examples of GTI are shown in Figure 1.

No significant difference between TDT patients and healthy subjects was found in terms of age and sex (*p* = 0.234 and *p* = 0.708, respectively).

**Figure 2:**
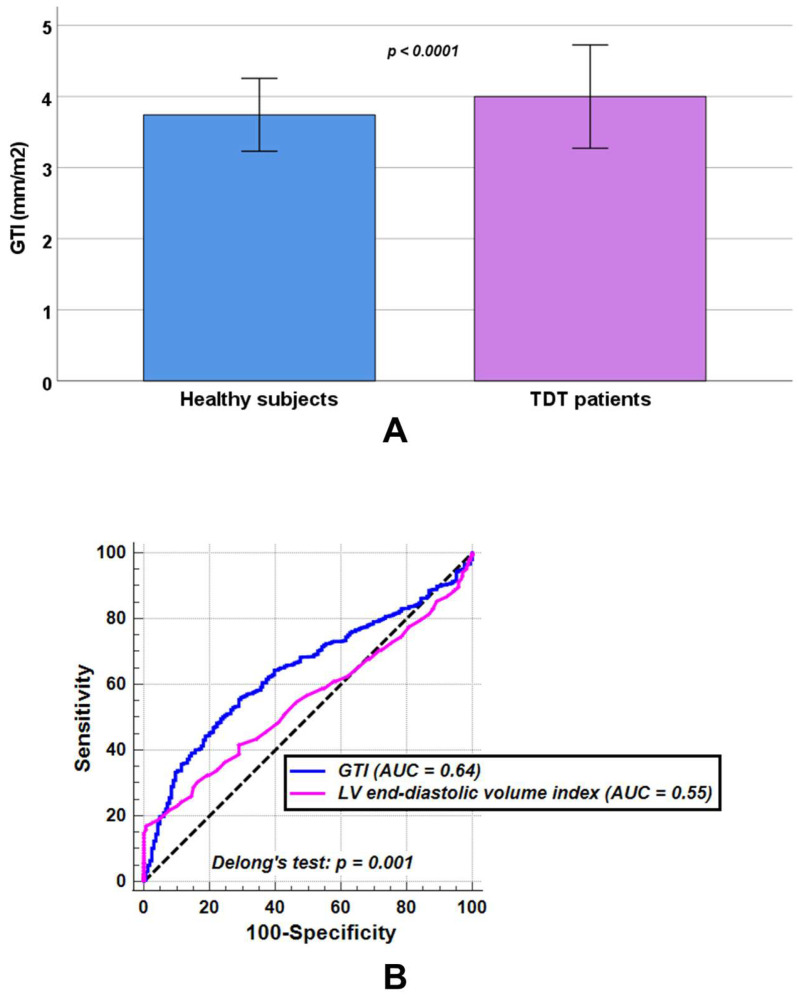
(**A**) Comparison of global wall thickness index (GTI) between healthy subjects and patients with transfusion-dependent thalassemia (TDT). (**B**) Receiver operating characteristic curve (ROC) curve analysis of GTI [blue] and left ventricular (LV) end-diastolic volume index [pink] to discriminate between TDT patients and healthy subjects. AUC = area under the curve.

In comparison with healthy individuals, patients with TDT showed significantly increased LV end-diastolic volume index (82.48 ± 16.96 mL/m^2^ vs. 78.86 ± 10.28 mL/m^2^, *p* = 0.041) and LV end-systolic volume index (31.57 ± 10.81 mL/m^2^ vs. 28.87 ± 6.35 mL/m^2^, *p* = 0.011), while no statistically significant differences were observed between the two groups in LV mass index (54.91 ± 13.69 g/m^2^ vs. 53.62 ± 8.92 g/m^2^, *p* = 0.484) or LV ejection fraction (62.32 ± 6.99% vs. 63.35 ± 5.36%, *p* = 0.097). TDT patients had a significantly higher GTI than healthy subjects (3.99 ± 0.73 mm/m^2^ vs. 3.74 ± 0.51 mm/m^2^, *p* < 0.0001) (Figure 2A).

At ROC curve analysis, a GTI > 3.91 mm/m^2^ discriminated between healthy subjects and TDT patients with a sensitivity of 55.9% and a specificity of 70.5% (*p* < 0.0001). The AUC was 0.64 (95% CIs: 0.61 to 0.67). An LV end-diastolic volume index > 98.00 mL/m^2^ discriminated between healthy subjects and TDT patients with a sensitivity of 16.8% and a specificity of 99.4% (*p* = 0.014). The AUC was 0.55 (95% CIs: 0.52 to 0.58). According to Delong’s test, GTI performed significantly better in discriminating healthy subjects from TDT patients than LV end-diastolic volume index (*p* = 0.001) (Figure 2B).

In healthy subjects, the GTI was comparable between males and females (3.75 ± 0.57 mm/m^2^ vs. 3.73 ± 0.47 mm/m^2^, *p* = 0.911) and was independent from age (R = −0.112, *p* = 0.151).

### 3.2. Associations Between Demographic/Clinical Characteristics and GTI in TDT

GTI was significantly higher in males than in females (4.06 ± 0.71 mm/m^2^ vs. 3.95 ± 0.73 mm/m^2^, *p* = 0.004), while its correlation with aging showed a trend toward statistical significance (R = −0.057, *p* = 0.053).

GTI was comparable between non-splenectomized and splenectomized patients (4.01 ± 0.80 mm/m^2^ vs. 3.99 ± 0.66 mm/m^2^, *p* = 0.774), and was not significantly correlated with age at start of regular transfusions (R = −0.031, *p* = 0.379), chelation starting age (R = −0.098, *p* = 0.094), mean serum hemoglobin levels (R = −0.016, *p* = 0.596), and mean serum ferritin levels (R = 0.029, *p* = 0.350).

Among the 1105 patients evaluated for diabetes mellitus, the condition was identified in 15.7% of cases. The GTI was significantly higher in patients with diabetes than in patients without diabetes (4.08 ± 0.75 mm/m^2^ vs. 3.97 ± 0.70 mm/m^2^, *p* = 0.037).

### 3.3. Associations Between GTI and Iron Overload in TDT

GTI was not correlated with MRI LIC values (R = 0.056, *p* = 0.057), pancreatic T2* values (R = −0.026, *p* = 0.373), global heart T2* values (R = 0.023, *p* = 0.433), or number of segments with T2* < 20 ms (R = 0.046, *p* = 0.116).

**Figure 3:**
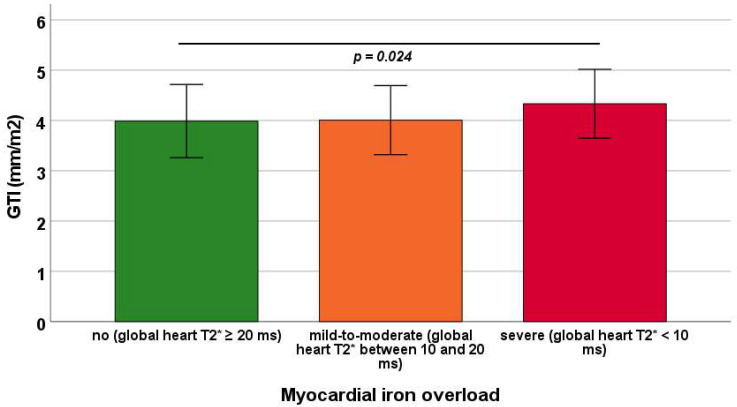
Mean GTI (global wall thickness index) in the three groups of transfusion-dependent thalassemia patients identified based on global heart T2* values. The horizontal line indicates a significant difference between two groups.

Mean GTI was 3.99 ± 0.73 mm/m^2^ in patients without MIO, 4.01 ± 0.69 mm/m^2^ in those with mild-to-moderate MIO, and 4.33 ± 0.68 mm/m^2^ in those with severe MIO. GTI was significantly increased in patients with severe MIO compared with those without MIO (*p* = 0.024), whereas other between-group comparisons did not reach statistical significance (Figure 3).

### 3.4. Predictors of GTI

Table 2 summarizes the results of the univariable and multivariable regression analyses for predictors of GTI. In the univariable models, male sex and severe MIO (compared with patients with moderate-to-mild or no MIO grouped together) were significantly associated with GTI, and both remained independent predictors in the multivariable analysis (F = 7.77, *p* < 0.0001). No evidence of collinearity was found between the two variables (tolerance = 0.99 and variance inflation factor = 1.01).

### 3.5. Association of GTI with LV Function and Fibrosis

GTI was not associated with LV ejection fraction (R = 0.021, *p* = 0.472).

Of the 366 (31.7%) patients who underwent contrast administration, 95 (26.3%) demonstrated replacement myocardial fibrosis in at least one myocardial segment. The majority exhibited a non-ischemic pattern of LGE (N = 91, 95.8%), with septal involvement observed in 81.1% of cases. GTI was comparable between patients without and with LGE (4.12 ± 0.61 mm/m^2^ vs. 4.17 ± 0.69 mm/m^2^, *p* = 0.899). Among LGE-positive patients, the mean number of affected myocardial segments was 2.6 ± 1.8 (range: 1–9), and 63 (66.3%) patients displayed multifocal involvement, defined as two or more affected segments. GTI was not correlated with the number of LGE-positive segments (R = 0.137, *p* = 0.186).

### 3.6. Association Between GTI and History of Heart Failure

Sixty-six patients had a history of heart failure.

**Figure 4:**
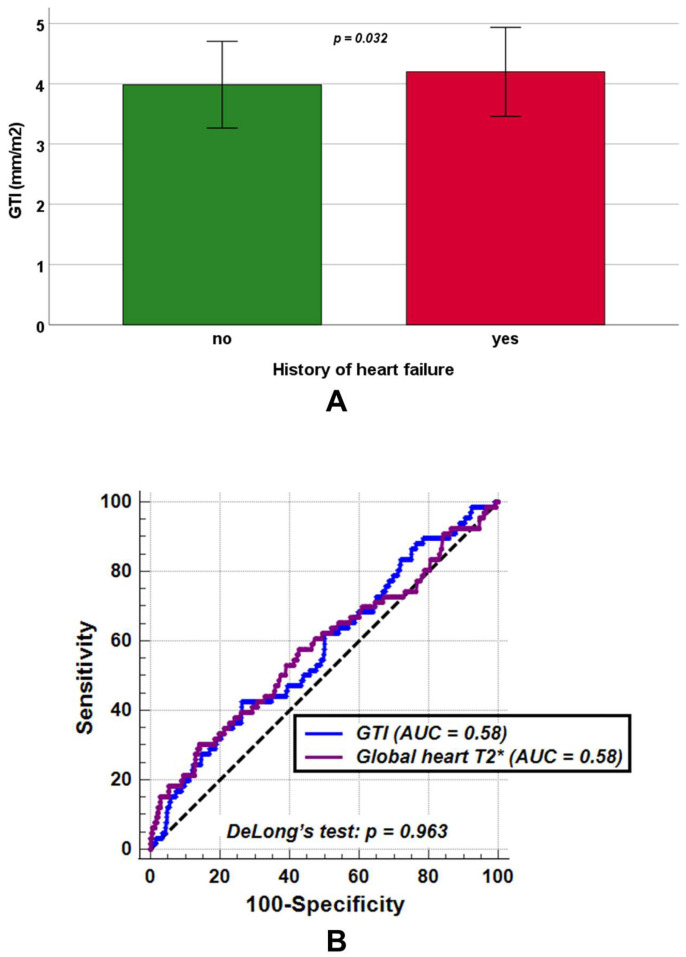
(**A**) Mean GTI (global wall thickness index) in transfusion-dependent thalassemia patients without and with a history of heart failure. (**B**) Comparison of receiver operating characteristic curve (ROC) curves for GTI [blue] and global heart T2* values [purple] to detect a positive history of heart failure. AUC = area under the curve.

No significant differences between patients without and with a history of heart failure were found in LV end-diastolic volume index (82.48 ± 16.90 mL/m^2^ vs. 81.58 ± 17.99 mL/m^2^, *p* = 0.761) and LV mass index (54.72 ± 13.76 g/m^2^ vs. 56.76 ± 13.12 g/m^2^, *p* = 0.081), while GTI was significantly increased among patients with a history of heart failure (4.19 ± 0.74 mm/m^2^ vs. 3.98 ± 0.72 mm/m^2^, *p* = 0.038) (Figure 4A).Moreover, patients with a history of heart failure exhibited significantly lower LV ejection fraction (59.77 ± 9.88% vs. 62.49 ± 6.76%, *p* = 0.030) and global heart T2* values (33.16 ± 13.17 ms vs. 37.28 ± 9.30 ms, *p* = 0.032).

ROC curve analysis demonstrated that a GTI > 4.34 mm/m^2^ predicted a positive history of heart failure with a sensitivity of 42.4% and a specificity of 73.7% (*p* = 0.041), yielding an AUC of 0.58 (95% CIs: 0.55 to 0.61). Similarly, a global heart T2* ≤ 29.56 ms predicted a positive heart failure history with a sensitivity of 30.3% and a specificity of 85.9% (*p* = 0.049), also with an AUC of 0.58 (95% CIs: 0.55 to 0.61). DeLong’s test revealed no significant difference between the two AUCs (*p* = 0.963) (Figure 4B). The ROC curve analysis did not identify an LV ejection fraction threshold that significantly improved the detection of a positive heart failure history with acceptable sensitivity and specificity (AUC = 0.55, 95% CI: 0.52 to 0.58, *p* = 0.223).

## 4. Discussion

In this large cohort of well-treated TDT patients, we explored the clinical significance of the GTI in comparison with healthy subjects, as well as its associations with demographic and clinical variables, iron overload, myocardial function, and heart failure.

GTI emerged as a more sensitive marker of LV remodeling in TDT than conventional volumetric or functional indices. While LV mass index and ejection fraction did not differ significantly between patients and healthy subjects, both LV end-diastolic volume index and GTI were consistently elevated in TDT. These findings support the concept that myocardial remodeling in TDT follows a stepwise course, with early geometric alterations detectable by CMR preceding overt systolic dysfunction or increases in LV mass [18,60,61]. ROC analysis demonstrated that GTI had superior discriminatory ability compared with LV end-diastolic volume index, highlighting its potential value in distinguishing TDT patients from healthy individuals. Although its diagnostic accuracy was modest (AUC 0.64), limiting its use as a standalone parameter, GTI appears to capture subtle myocardial changes not reflected by conventional indices, underscoring that standard measures of systolic function may underestimate early cardiac involvement in this population.

In TDT, male sex emerged as a consistent predictor of higher GTI, both in univariable and multivariable analyses. In contrast, GTI was comparable between sexes in our healthy controls. A previous CMR study reported significantly higher absolute GT values in healthy males compared to females (7.2 ± 0.7 mm vs. 5.9 ± 0.6 mm) [46]. However, indexing for BSA reduced this difference, making GTI less sex-dependent (females: 3.4 ± 0.4 mm/m^2^ vs. males: 3.6 ± 0.4 mm/m^2^), suggesting that BSA adjustment compensates for sex-related differences in cardiac morphology and provides a standardized measure suitable for clinical practice. Several factors may explain the association between male sex and higher GTI in TDT. One possibility is the relative under-transfusion in males, as current transfusion protocols do not adjust for gender-related differences in blood volume [62]. This may result in a more chronically anemic state, contributing to volume overload and ventricular remodeling. Another potential mechanism is the differential susceptibility to oxidative stress: despite similar cardiac iron levels, females may tolerate chronic oxidative stress more effectively [63,64], reducing the impact of iron toxicity on myocardial structure over time.

Although GTI was not significantly correlated with global heart T2* values, severe MIO emerged as an independent predictor of higher GTI, indicating that extreme iron deposition can drive maladaptive left ventricular remodeling. The lack of a direct association with current T2* values is likely influenced by prior CMR-guided adjustments in chelation therapy, as most patients were not naïve to CMR imaging. While intensive chelation can effectively reduce myocardial iron overload [65,66,67,68,69,70], structural changes induced by previous iron accumulation may be only partially reversible. Consequently, GTI may reflect the cumulative or residual effects of past iron burden on LV geometry rather than the contemporaneous iron load alone. The observation that GTI increases primarily in patients with severe MIO further underscores the progressive nature of iron-related cardiac remodeling, suggesting a threshold effect in which only substantial iron overload produces measurable structural alterations. These findings highlight the importance of early detection and aggressive iron management to prevent irreversible myocardial remodeling and potential progression to cardiomyopathy.

The lack of an association between GTI and LV ejection fraction suggests that early geometric remodeling captured by GTI can occur independently of global systolic function. Similarly, the absence of a relationship between GTI and replacement myocardial fibrosis indicates that GTI primarily reflects diffuse or early structural remodeling rather than an established focal scar. However, this result should be interpreted with caution, as fibrosis assessment by LGE was available for only a third of the cohort, which may have affected the statistical power of the analysis.

Our findings show that GTI was significantly higher in TDT patients with a history of heart failure, accompanied by reduced LVEF and impaired myocardial iron status. The relatively modest sensitivity of GTI in predicting heart failure history likely reflects both the limited number of positive cases and the multifactorial pathogenesis of heart failure in thalassemia. Importantly, GTI demonstrated a diagnostic performance comparable to global myocardial T2* and showed higher sensitivity and specificity than LV ejection fraction. From a pathophysiological perspective, our results are clinically meaningful. LV ejection fraction is a conventional measure of systolic performance [38,71], but in thalassemia it is heavily influenced by chronic anemia and a compensatory hyperdynamic circulation, which maintains elevated stroke volumes and ventricular dimensions [18,21]. As a result, LV ejection fraction often remains within normal or near-normal ranges, even in the presence of impaired myocardial shortening and evolving systolic dysfunction [72,73]. This limitation reduces its utility for early risk stratification. In contrast, GTI captures changes in myocardial geometry and deformation that are more closely linked to subtle alterations in ventricular structure. Thus, elevations in GTI may reflect the onset of adverse remodeling well before declines in LV ejection fraction become evident.

Taken together, our results suggest that GTI represents a sensitive marker of early myocardial involvement and may serve as a valuable tool to improve the clinical management of patients with TDT. By identifying adverse remodeling at a subclinical stage, GTI could complement established markers such as T2*, offering an opportunity for earlier intervention and potentially better long-term outcomes.

### Limitations

This work has several important constraints.

The retrospective nature of the study introduces the possibility of selection bias and incomplete clinical information, with some relevant variables not captured in the database. Advanced CMR mapping techniques such as T1, T2, and extracellular volume quantification were not available at the time of patient enrollment, limiting our ability to perform a complete and sensitive myocardial tissue characterization [74,75,76]. Similarly, myocardial deformation indices were not evaluated. Although strain analysis is increasingly recognized as a sensitive marker of subclinical ventricular dysfunction [77,78], the lack of uniform access to feature tracking software across E-MIOT centers prevented its systematic inclusion.

The cross-sectional design restricts interpretation of causality and precludes conclusions on the temporal evolution of GTI in relation to disease progression. The prognostic significance of GTI in anticipating adverse cardiovascular outcomes was not explored, and longitudinal changes in this parameter remain undefined. Future prospective studies with serial follow-up are required to establish clinically relevant GTI thresholds and to clarify whether its integration with conventional indices, such as myocardial T2*, enhances risk stratification.

## 5. Conclusions

In patients with TDT, GTI is significantly elevated compared with healthy controls, with male sex and severe myocardial iron overload emerging as its main determinants. Increased GTI is linked to a history of heart failure, suggesting that it may reflect early adverse remodeling and could complement established markers such as myocardial iron load and LV function. Although not sufficient as a standalone tool, GTI shows promise as part of a multiparametric approach to improve risk stratification and guide management in TDT, a role that should be confirmed in prospective studies.

## Figures and Tables

**Table 1:** Demographic, clinical, and MRI data of TDT patients.

Variable	TDT Patients (N = 1154)
Females, N (%)	611 (52.9)
Age (years)	37.46 ± 10.67
Transfusion starting age (months)	14.98 ± 15.37
Chelation starting age (years)	5.23 ± 6.05
Splenectomy, N (%)	650 (56.3)
Pre-transfusion hemoglobin (g/dL)	9.66 ± 0.53
Serum ferritin (ngm/L)	1062.43 ± 1325.07
Diabetes, N (%)	174/1105 (15.7)
MRI LIC (mg/g dw)	6.38 ± 9.73
Global pancreas T2* (ms)	12.76 ± 10.35
Global heart T2* (ms)	37.08 ± 9.61
MIO, N (%)	
no	1055 (91.4)
moderate-to-mild	64 (5.6)
severe	35 (3.0)
Number of segments with T2* < 20 ms	1.58 ± 4.19
LV end-diastolic volume index (mL/m^2^)	82.48 ± 16.96
LV end-systolic volume index (mL/m^2^)	31.57 ± 10.81
LV stroke volume index (mL/m^2^)	51.39 ± 10.58
LV mass index (g/m^2^)	54.91 ± 13.69
LV ejection fraction (%)	62.32 ± 6.99
Replacement myocardial fibrosis, N (%)	95/366 (26.0)
GTI (mm/m^2^)	3.99 ± 0.73

TDT = transfusion-dependent thalassemia, N = number, MRI = magnetic resonance imaging, LIC = liver iron concentration, MIO = myocardial iron overload, LV = left ventricular, GTI = global wall thickness index.

**Table 2:** Determinants of GTI identified by univariable and multivariable linear regression analyses.

	Univariable Regression		Multivariable Regression	
	B (95% CIs)	*p*-Value	B (95% CIs)	*p*-Value
Male sex	0.111 (0.027 to 0.195)	0.009	0.119 (0.036 to 0.203)	0.005
Age > 75th percentile	0.021 (−0.076 to 0.118)	0.671		
Splenectomy	−0.017 (−0.102 to 0.067)	0.687		
Diabetes	0.106 (−0.010 to 0.222)	0.072		
Severe MIO	0.344 (0.100 to 0.588)	0.006	0.367 (0.123 to 0.611)	0.003

CIs = confidence intervals; MIO = myocardial iron overload.

## Data Availability

The data presented in this study are available on request from the corresponding author due to privacy.
